# The architecture of the European Union’s pandemic preparedness and response policy framework

**DOI:** 10.1093/eurpub/ckac154

**Published:** 2022-11-18

**Authors:** Dimitri Eerens, Rok Hrzic, Timo Clemens

**Affiliations:** European Public Health Programme, Department of International Health, Care and Public Health Research Institute (CAPHRI), Faculty of Health, Medicine, and Life Sciences, Maastricht University, Maastricht, The Netherlands; Department of International Health, Care and Public Health Research Institute (CAPHRI), Faculty of Health, Medicine, and Life Sciences, Maastricht University, Maastricht, The Netherlands; Department of International Health, Care and Public Health Research Institute (CAPHRI), Faculty of Health, Medicine, and Life Sciences, Maastricht University, Maastricht, The Netherlands

## Abstract

**Background:**

COVID-19 has highlighted the importance of preparedness and response systems when faced with a pandemic. The rapid spread of the disease throughout Europe raised questions about the capacity of the European Union (EU) and its Member States to combat serious cross-border threats to health. This article provides an overview of institutional arrangements for pandemic preparedness before the COVID-19 pandemic and outlines the changes proposed by the European Health Union (EHU) framework.

**Methods:**

A systematic review of relevant EU law, EU policy documents and the scientific literature was conducted. EUR-lex, PubMed, Web of Science core collection and Google Scholar databases were searched for relevant records published after the year 2000. The proposed new regulatory framework was extracted from the EHU legislative package. The results were organized according to the Public Health Emergency Preparedness Logic Model.

**Results:**

The main EU bodies involved in preparedness and response are the European Centre for Disease Prevention and Control (ECDC), the European Commission and the Health Security Committee (HSC). The proposed changes of the EHU focus on strengthening the auditing capabilities of the ECDC, increasing the scope of EU action in managing medical countermeasures, and further formalizing the HSC.

**Conclusions:**

The proposal takes bold steps to address technical and political issues of preparedness and response; whereas, on the latter point, it is likely that amendments to the proposal will not address long-standing challenges in preparing for and coordinating national responses to a future EU-wide pandemic.

## Introduction

As Europe became the epicenter of the COVID-19 pandemic in March 2020, it became clear that it was not fully prepared to deal with a cross-border health threat.^1^ By 18 March 2020, more than 250 million people in Europe were in lockdown.^1^ While European Union (EU) Member States struggled to control their national epidemiological situation, the public’s attention focused as well on EU actions to combat the pandemic.

While EU policy on health security and health threat preparedness is evolving, it did not emerge recently. The history of European health security cooperation already stretches back to the late 90s, when structures for communicable disease control were already established in the EU through Decision 2119/98/EC which provided for a European network for epidemiological surveillance and disease control. This was further complemented by the establishment of the European Centre for Disease Prevention and Control (ECDC) in 2004 that was tasked with strengthening Europe’s defense against communicable diseases. Under the Treaty of Lisbon, the EU has a mandate to protect human health against serious cross-border threats according to Article 168.^2^ The 2013 Council decision on serious cross-border threats to health (SCBTH) was the last addition to EU’s framework governing health threat preparedness and response in the EU prior to the COVID-19 outbreak in Europe.

However, the large negative impact of the COVID-19 pandemic on the EU has called into question whether the patchwork of health emergency preparedness legislation was fit for purpose^3^ and prompted proposals for revisions as part of the European Health Union (EHU) initiative (see table 1). Already in 2016, a review of the EU’s health security framework revealed six key gaps.^4^ It emphasized the existence of overall weaknesses in preparedness coordination including at the EU level as well as gaps in ECDC generic preparedness capacities. Moreover, it identified that the Health Security Committee (HSC) faced various challenges limiting the full use of its mandate, as well as coordination of response rules being generally difficult to observe in practice.

**Table 1:** European Health Union legislative proposals

Proposed legislation
Proposal for a regulation on serious cross-border threats to health and repealing Decision 1082/2013/EU
Proposal for a regulation amending Regulation (EC) No 851/2004 establishing a European Centre for Disease Prevention and Control
Proposal for a regulation on a reinforced role of the European Medicines Agency in crisis preparedness and management for medicinal products and medical devices
Council regulation on a framework of measures for ensuring the supply of crisis-relevant medical countermeasures in the event of a public health emergency at Union level
Commission decision establishing the Health Emergency Preparedness and Response Authority

Research in pandemic preparedness has long focused on low- and middle-income countries with a view of improving preparedness and response capabilities,^5^ whereas the literature on European health security frameworks is sparse. Existing studies have focused on preparedness for existing threats such as influenza^6^ and not a wider range of health threats such as novel infectious diseases. Despite this, the literature has highlighted the significant heterogeneity in pandemic preparedness across EU Member States and the coordination challenges this represents for the EU.^6^ This article contributes to this literature in two ways. First, we seek to review the institutional arrangements for pandemic preparedness and response in the EU using the Public Health Emergency Preparedness (PHEP) model^7^ as an organizing framework. We define institutional arrangements as the policies, rules, legislation and norms that the European institutions observe when crafting an EU-wide pandemic preparedness framework. Second, we analyze the changes proposed in the EHU initiative with a view of identifying amendments addressing the previously identified inadequacies, particularly focusing on the role of the ECDC and the HSC in pandemic preparedness and preventing shortages of medical countermeasures (MCMs). This article is divided into several sections, comprising Methods, Results, Discussion and Conclusion. In the Results section, the preparedness and response capabilities of the EU will first be described in pre-2020 arrangements and then subsequently the changes introduced by the proposed legislation will be highlighted.

## Methods

A policy document review of relevant EU law, EU policy documents was conducted. EU treaties, regulations and decisions were reviewed to map out the EU institutions and other actors involved in pandemic preparedness and response using the Stoto *et al*.^7^ PHEP model. Where possible, scientific literature on EU health threat preparedness was consulted to validate the interpretation of the policy documents.

### Eligibility criteria

We considered primary and secondary EU legal sources, EU reports and peer-reviewed scientific articles as eligible if they were written in English and published in the year 2000 or later. The year 2000 was selected as a cutoff because it was only in 1998 that the decision on a European network for epidemiological surveillance and control was adopted by the European Parliament and Council.^8^ Moreover, the analysis of this article is focused on existing arrangements as well as proposed arrangements, whereas previous informal arrangements may have been partly subsumed into existing legislation (e.g. 2119/98EC being repealed by Decision 2013/82). National Member State policy documents were not included as national arrangements for pandemic preparedness are not the focus of this article. Policy documents were considered eligible if they contained information directly related to preparedness for pandemics and health threats at the European Level. Documents relating to bioterrorism were excluded from the analysis.

### Search strategy

EU treaties, regulations and decisions were obtained through the legal database EUR-lex. Policy documents were obtained by hand searching the European Commission’s online portal on health security, the websites of the HSC, the EU Civil Protection and Humanitarian Aid Operations Directorate General, the European Parliamentary Research Service and the European Center for Disease Prevention and Control. PubMed, Google Scholar and Web of Science core collection databases were queried for scientific articles and legal commentary on pandemic preparedness in the EU. Reference lists of included documents were reviewed for additional relevant literature.

### Selection and extraction

For all uncovered documents, titles and abstracts of the articles were screened for relevance. In the next step, the full text of the documents was reviewed against eligibility criteria and included if all conditions were met. The primary screening was performed by D.E. In ambiguous cases, the authors reviewed the full text of the documents in question and came to a unanimous decision. Once documents were identified and selected (see Supplementary figure S1), the authors reviewed the information on the competences and capabilities of the EU with reference to the framework of the PHEP model. Information on the proposed changes to the pandemic preparedness policy framework were extracted by comparing the existing legislation with the EHU initiative. The innovations of the proposed legislation were then organized into a matrix and classified according to the PHEP model (see Supplementary materials S1 and S2). Where possible, peer-reviewed scientific articles were used to validate the authors’ interpretation.

### The Public Health Emergency Preparedness model

The Stoto *et al*.^7^ PHEP model was designed to help EU Member States identify their preparedness shortcomings. The authors define PHEP as

the capability of the public health and health care systems, communities and individuals to prevent, protect against, quickly respond to, and recover from health emergencies, particularly those whose scale and timing, or unpredictability threatens to overwhelm routine capabilities (Stoto *et al*.,^7^ p. 475).

The model distinguishes between capacities and capabilities, with capacities referring to infrastructure, trained personnel, policies and procedures and capabilities referring to the use of capacities to effectively identify, characterize and respond to emergencies. This framework is used as a tool to categorize facts and is not a normative proposal. There exist other frameworks measuring pandemic preparedness such as the Joint European Pandemic Preparedness Self-assessment Indicators^9^ or the Joint external evaluation tool: International Health Regulations (2005),^10^ which provide a normative prescription. The scope of this study does not focus on the effectiveness of preparedness and response arrangements but rather on mapping out the EU institutional architecture in pandemic preparedness. The PHEP model has the additional advantage that it does not narrowly focus on preparedness for specific health threats such as influenza pandemics but can be applied broadly to categorize elements of preparedness across a spectrum of pandemic health threats.

## Results

**Figure 1:**
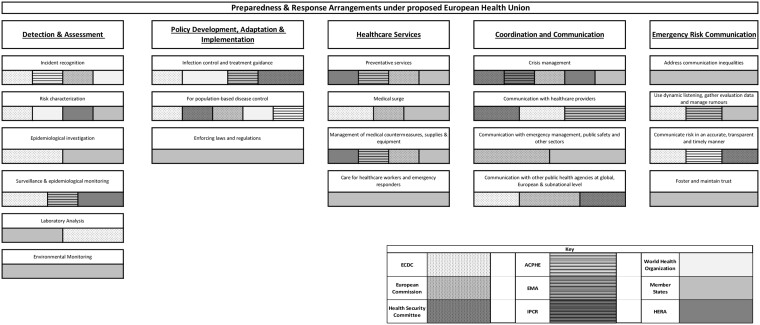
Preparedness and response arrangements under the proposed European Health Union

**Figure 2:**
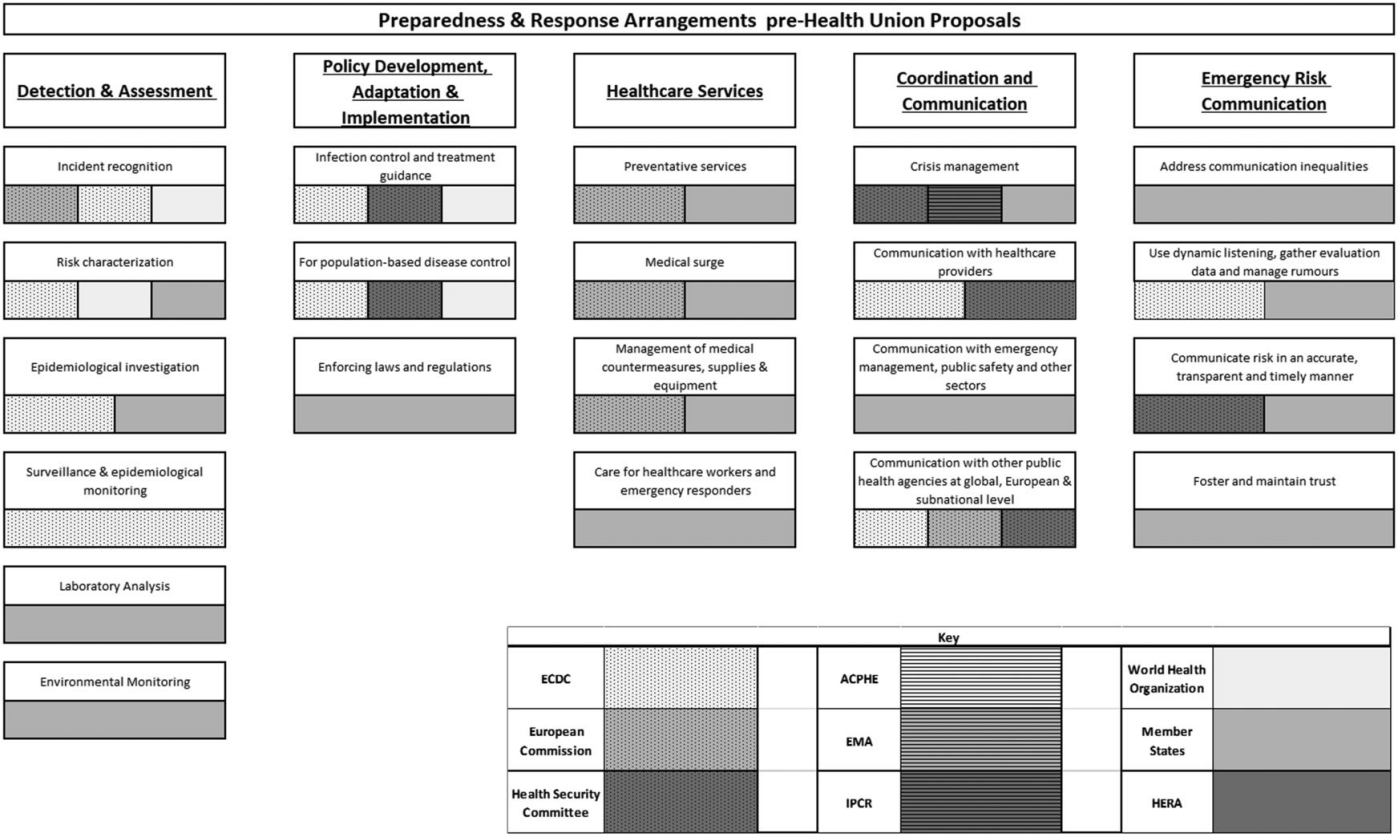
Preparedness and Response Arrangements pre-Health Union proposals

Six existing or proposed pieces of EU legislation, 10 other policy documents (e.g. briefings, reports, communications and expert opinions), and 15 journal articles, were included in the review. This corpus was used to map the various institutions and bodies involved in preparedness and response planning according to the PHEP model (figure 1). Additional detail on each of the capabilities is provided subsequently. The bars reflect the general presence and competence of identified bodies in the preparedness and response capabilities and do not reflect the degree of influence they hold. The existing EU health preparedness framework prior to the introduction of the European Health Union proposals can be identified in figure 2.

### Detection and assessment

The EU holds capabilities in Detection and Assessment, with the ECDC being tasked with identifying and assessing current and emerging threats to human health from communicable disease.^11^ Primarily, ECDC collects, analyses and disseminates surveillance on communicable diseases.^12^^,^^13^ While the ECDC uses a variety of tools and data sources to monitor disease outbreaks and emerging threats, it remains dependent on Member States for delivering data. Upon Member State request, the ECDC can mobilize investigation and public health teams^11^ but this is rarely feasible as the mobilization of these teams requires substantial financial and human resources.^14^^,^^15^ The ECDC also encourages cooperation between laboratories across the EU to foster the development of capacity for diagnosis, detection and characterization of infectious agents but ECDC itself does not have laboratory facilities.^16^^,^^17^

The proposed regulation on SCBTH promises to improve EU efforts in surveillance and epidemiological monitoring through the specification of Member States’ obligations on epidemiological surveillance reporting. Through the epidemiological surveillance network, Member States are to provide information on molecular pathogen data, health systems data and contact tracing monitoring systems. The European Medicines Agency (EMA) and the Health Emergency Preparedness and Response Authority (HERA) would play a role in epidemiological surveillance with a view of identifying potential medicine shortages. An Advisory Committee on Public Health Emergencies (ACPHE) would be established, advising the Commission whether a threat constitutes a public health emergency at the Union level. The regulation extends the effect of a recognition of a public health emergency at the Union level to allow for the use of medicines with limited clinical information or effectiveness, activating mechanisms to monitor shortages of medical countermeasures (MCMs) as well as launch processes to address them. Finally, the ECDC would oversee the operation of the network of EU reference laboratories. These reference laboratories would provide support to national reference laboratories to promote good practice and alignment on diagnostics, testing methods, and notification and reporting of disease by Member States. While these provisions preclude the establishment of a physical EU reference laboratory, they promote action on improving the EU’s capability for laboratory analysis.

### Policy development

Decision 1082/2013 and the World Health Organization (WHO) 2005 International Health Regulations oblige EU Member States and signatories to create and maintain adequate pandemic preparedness plans.^18^ The ECDC and the Commission can produce non-binding policy recommendations and guidelines, while national governments retain the responsibility to decide on the implementation of health measures and policies.^19^ Nonetheless, ECDC outputs are frequently used in decision-making at the EU level as well as at the national level.^15^

Under Article 5 of the proposed regulation on SCBTH, the new Union pandemic plan would include arrangements for cooperation on governance, capacities and resources on a variety of issues such as epidemiological surveillance, early warning and risk assessment. Significantly, the proposals confer upon the ECDC the power to audit Member States’ implementation of preparedness and response plans as well as their coherence with the EU preparedness plan. The HSC and the ECDC would be able to adopt non-binding recommendations on response to a health crisis and a new advisory committee would provide advice on the formulation of response measures and the identification of inconsistencies in clinical management and treatment.

### Healthcare services

The EU cannot deploy and manage health services, but it assists national services through EMA accelerating the authorization procedure for medicines and countermeasures. The EU can also deploy assistance and medical personnel through the Union Civil Protection Mechanism (UCPM), which operates on a voluntary opt-in basis.^12^^,^^19–21^ To further strengthen the EU’s disaster preparedness, the Commission created rescEU, a strategic medical reserve and distribution mechanism operating under the direction of the UCPM.^19^^,^^20^ The EU also has some medical surge capabilities through the European Medical Corps, functioning as a pool of medical response capacities committed by Member States to be used when the national health system capacity is overwhelmed.^19^^,^^22^

The proposed regulations on SCBTH and the ECDC pave the way for wider ambitions of EU Healthcare Services capabilities. First, the Union preparedness and response plan could help in better allocating resources and capacities across Member States in emergency situations.^23^ Second, the prohibition of parallel negotiation processes for MCMs during the Joint Procurement Procedure would strengthen the influence of the Commission in the management of MCMs. Third, a new ‘EU Health Taskforce’ (EUHTF) would assist Member States’ response to outbreaks of communicable diseases.^24^ Members of this task force would also work closely with Ministry of Health officials in long-term capacity building to strengthen the implementation of the 2005 WHO IHR. Fourth, EMA’s extended mandate would allow it to provide recommendations to address medicine shortages and to accelerate the provision of advice for clinical trials targeting diseases with pandemic potential. Fifth, a new Commission structure, HERA, would become responsible for promoting R&D of MCMs, stockpiling MCMs and reserving manufacturing capacities to rapidly scale up the production of MCM’s during a health crisis.^19^

### Coordination and communication

Member States lead the coordination of responses to health threats through structures such as the HSC and the Integrated Political Crisis Response (IPCR).^12^^,^^25^ Moreover, Member States coordinate their risk and crisis communication for the public as well as healthcare professionals through the HSC.^12^ While Decision 1082/2013/EU sets the expectation that Member States should inform each other prior to the implementation of their response measures, there is no explicit mechanism to sanction Member States failing to do so.^14^

The most significant change under the proposed regulation on SCBTH is the further formalization of the role of the HSC in health emergencies, which would adopt opinions and guidance to create a coordinated EU response to cross-border threats to health.^23^ The responsibility of the HSC would be to promote the interoperability of national plans as well as the intersectoral dimension of preparedness and response planning is also novel. In general, the language regarding the activities of the HSC is stronger, specifically stating that Member States shall *coordinate* within the HSC, instead of *consult* as in the Decision 1082.

### Emergency risk communication

Risk and crisis communications are currently coordinated within the HSC with a view to adapting such communications to Member States needs and circumstances.^19^^,^^26^ The HSC’s Communicators network provides crisis communication expertise and guidance as part of a strategy for the management of cross-border health threats.^27^ Under the current legislative framework, the ECDC collaborates with Member States^11^ and the Commission to promote coherence in risk communication.

Under the new SCBTH regulation, the EU preparedness and response plan would include arrangements for risk and crisis communications. This is further supported by Member State reporting requirements including the disclosure of risk communication capacities. The advisory committee would also provide advice on response including advice on risk and crisis communication to be addressed to all Member States. Under the new mandate of the ECDC, the agency would also be tasked with providing the HSC with evidence-based communication messages to the public. Despite the revised SCBTH and ECDC regulations, neither proposals outline substantive action to be taken on developing Emergency Risk Communication capabilities such as addressing communication inequalities, counteracting misinformation and fostering and maintaining trust. Here, the EMA regulation fills a gap as it also contains several provisions regarding communication of the activities of the steering groups and ETF to the general public as well as interested parties. Interestingly, the regulation also tasks the EMA with responding to disinformation targeting the work of the steering groups.^28^

## Discussion

This article reviewed the institutional arrangements for pandemic preparedness and response in the EU and the changes proposed under the EHU initiative. Overall, the proposed changes would provide an increase in EU detection and assessment capabilities, policy development and implementation and the management of MCMs. The innovations include an EU-wide pandemic preparedness plan, expanding the ECDC’s mandate to include auditing Member States and establishing an EU Health taskforce, and improving the capacity of the Union to develop, stockpile and distribute MCMs via HERA and EMA.

The most significant change to the ECDC regulation is allowing the agency to audit Member States and gather data on national health systems to provide timely recommendations on the management of health threats. This is important because some EU Member States fall short of the implementation of the International Health Regulations.^29^ In addition, the ECDC is conferred with a maintained capacity to carry out missions to Member States to provide recommendations on response through the EUHTF. This could be helpful in strengthening Member State’s preparedness as well as assisting with the response to health threats, of which both vary substantially across the EU.^29^ Previously the provisioning of expert assistance and the coordination of investigation teams was explicitly limited to the financial capacity of the center. The new legislation specifically enables the ECDC to establish the taskforce as well as maintain the capacity to carry out missions, further supporting the agency in creating a broader financial scope to support its expanded responsibilities. However, with regards to the review of Member States preparedness level, Council of the EU negotiation mandates aim to replace the term audit with examine, weakening the ECDC’s powers in holding Member States accountable.^30^ Another obstacle is the lack of adequate funding to accompany the increase in ECDC’s responsibilities.^29^^,^^31^

The most significant innovation of the proposed regulation on SCBTH is the establishment of an EU-wide pandemic preparedness plan. The regulation would also further extend the EU’s capabilities in healthcare services, especially concerning the management of MCMs. The proposed prohibition of parallel negotiations during the joint procurement process would further strengthen the bargaining power of the Commission and better coordination. In the stockpiling and distribution of MCMs, instruments such as the UCPM were unable to effectively address situations where health crises occur simultaneously in all Member States.^20^^,^^29^^,^^32^ HERA and the EMA have been granted the power to anticipate shortages of MCMs, with HERA being able to direct R&D funding to produce new countermeasures as well as reserve manufacturing capacity to rapidly scale up production of countermeasures during a pandemic. However, questions may still be raised regarding the uneven participation from Member States, as research has identified varying levels of resources as well as asymmetries in technical capacity of national disease control agencies and an uneven distribution of health professionals across EU countries^8^^,^^13^^,^^33^ Future research will need to further investigate to what extent differing national priorities and resources affect the coherence of a European preparedness plan and the implementation of the regulatory changes.

Member States remain reluctant to surrender their competence in the risk and response management. The proposals provide the HSC with the ability to adopt opinions and guidance, which could help coordinating a European response, as during the 2009 H1N1 pandemic, decisions taken by the HSC were perceived as de-facto binding by the other Member States.^34^ However, others favor a broadened mandate of the HSC through the acquisition of extended coordinating powers when a state of emergency is triggered.^14^ Member States with the assistance of the Commission and the ECDC would negotiate binding coordination measures allowing for Member States to preserve decision-making powers while providing a temporary mechanism of binding coordination.^14^^,^^31^ However, the current proposal does not include such provisions. It remains to be seen whether such recommendations would be taken up by Member States.

### Strengths and limitations

This article has several limitations. Researching the effectiveness of existing arrangements in practice is beyond the scope of this article’s research questions and is different to examining the mandate and mission of each EU body. This article addressed this gap by incorporating the analysis of policy documents such as parliamentary briefings, staff working documents and audit reports to fully uncover the role of various EU bodies and mechanisms in preparedness and response. The application of the PHEP model in this analysis is a significant advantage as other frameworks only narrowly understand in relation to specific disease threats such as influenza. The model enables us to take a macro-level perspective and identify the distribution of competences and capabilities between the EU and Member States with respect to a variety of different health threats. While this article has focused on the distribution of capabilities under the proposed regulations, further research can build upon this foundation by comparing the differences between the Commission proposals and the eventually adopted legislation.

## Conclusion

The EU mostly plays a supportive role in pandemic preparedness and response, and crisis management largely remains a competence of Member States. It is still too early to assess if the EHU will effectively address the shortcomings of the existing regulatory framework. Overall, it is likely that the changes will give the EU increased capabilities through a reinforced ECDC, HSC and joint procurement program, but the actual ability of the EU to directly intervene and manage future pandemics will be limited by the preferences of Member States.

## Supplementary data

Supplementary data are available at *EURPUB* online.

*Conflicts of interest*: None declared.

## Supplementary Material

ckac154_Supplementary_Data

## Data Availability

The data underlying this article are available in the article and in its online supplementary material. Public Health Emergency Preparedness model is useful in understanding European Union (EU) preparedness arrangements. Auditing the preparedness of EU Member States by the European Centre for Disease Prevention and Control (ECDC) would help to strengthen national preparedness plans and ensure consistency with the proposed EU pandemic preparedness plan. An extension of the mandate of the ECDC and its scope of activities should be accompanied by an increase in funding commensurate with its portfolio of action. Further research and attention should be directed at identifying if the ECDC is able to use the limited allocated resources to make effective use of the EU Health Taskforce.
