# Supplementary material for: The architecture of the European Union’s pandemic preparedness and response policy framework
Source: Eur J Public Health. 2022 Nov 18;33(1):42–8. doi: 10.1093/eurpub/ckac154 (PMC9898003; doi:10.1093/eurpub/ckac154)
Supplement: ckac154_Supplementary_Data [file ckac154_supplementary_data.zip › ckac154_Supplementary_Data/ejph-2022-06-om-0333-File006.docx]

Classification of Legislation / Legislative Proposals

Contents

[Proposal for a Regulation on serious cross-border threats to health 2](#_Toc106358142)

[Proposal for a Regulation amending Regulation No 851/2004 establishing a ECDC 45](#_Toc106358143)

[Regulation (EU) 2022/123 on reinforced role for the EMA in crisis preparedness and management for medicinal products and medical devices 60](#_Toc106358144)

[Proposal for Council Regulation on a framework of measures for ensuring the supply of crisis-relevant medical countermeasures in the event of a public health emergency at Union level 75](#_Toc106358145)

# Proposal for a Regulation on serious cross-border threats to health

*Supplementary Table 1.  Analysis of Proposal for a Regulation on serious cross-border threats to health and repealing Decision No 1082/2013/EU and classification of provisions into Public Health Emergency capabilities according to Stoto et al (2017) PHE logic model.*

| **Detection and Assessment** | **Policy Development, Adaptation and Implementation** | **Healthcare Services** | **Coordination and Communication** | **Emergency Risk Communication** |
| --- | --- | --- | --- | --- |
| **Article 4**    **4. The HSC shall be chaired by a representative of the Commission. The HSC shall meet at regular intervals and whenever the situation requires, on a request from the Commission or a Member State.**  **[ Incident recognition ]** | **Article 2**  **6. Member States shall retain the right to maintain or introduce additional arrangements, procedures and measures for their national systems in the fields covered by this Regulation, including arrangements provided for in existing or future bilateral or multilateral agreements or conventions, on condition that such additional arrangements, procedures and measures do not impair the application of this Regulation.**  **[Enforcing laws and regulations and Policy development]** | **Article 5**    **4.The Union preparedness and response plan shall include interregional preparedness elements to establish coherent, multi-sectoral, cross-border public health measures, in particular considering capacities for testing, contact tracing, laboratories, and specialised treatment or intensive care across neighbouring regions. The plans shall include preparedness and response means to address the situation of those citizens with higher risks.**    **[ Medical surge]** | **Article 2**    **4. In exceptional emergency situations, a Member State or the Commission may request the coordination of response within the HSC as referred to in Article 21, for serious cross-border threats to health other than those referred to in Article 2(1), if it is considered that public health measures taken previously have proven insufficient to ensure a high level of protection of human health.**  **[Crisis management]** | **Article 4**    **2. The HSC shall have the following tasks:**      **(c)coordination in liaison with the Commission of the risk and crisis communication and responses of the Member States to serious cross-border threats to health, in accordance with Article 21;**    **[ Communicate risk in an accurate, transparent and timely manner]** |
| **Article 5**  **3. The Union preparedness and response plan shall, in particular, include arrangements for governance, capacities and resources for:**    **c.  Epidemiological surveillance and monitoring**  **d.  Early warning and risk assessment**  **[ Surveillance and epidemiologic monitoring and Risk characterization]** | **Article 4**  **2.The HSC shall have the following tasks:**  **(d)adoption of opinions and guidance, including on specific response measures for the Member States for the prevention and control of serious cross-border threats to health.**  **[ For Population-based disease control and Enforcing laws and regulations ]** | **Article 12**  **1. The Commission and any Member States which so desire may engage in a joint procurement procedure conducted pursuant to Article 165(2) of Regulation (EU, Euratom) 2018/1046 of the European Parliament and of the Council 29 with a view to the advance purchase of medical countermeasures for serious cross-border threats to health.**    **[ Management of medical countermeasures, supplies and equipment ]** | **Article 2**  **5. The Commission shall, in liaison with the Member States, ensure coordination and information exchange between the mechanisms and structures established under this Regulation and similar mechanisms and structures established at Union level or under the Euratom Treaty whose activities are relevant for preparedness and response planning, monitoring, early warning of, and combating serious cross-border threats to health.**  **[Crisis management]** | **Article 5**  **3. The Union preparedness and response plan shall, in particular,  include arrangements for governance, capacities and resources for:**    **e.  The risk and crisis communication**  **[ Communicate risk in an accurate, transparent and timely manner]** |
| **Article 5**  **4. The Union preparedness and response plan shall include interregional preparedness elements to establish coherent, multi-sectoral, cross-border public health measures, in particular considering capacities for testing, contact tracing, laboratories, and specialised treatment or intensive care across neighbouring regions. The plans shall include preparedness and response means to address the situation of those citizens with higher risks.**  **[ Laboratory analysis and Epidemiologic investigation]** | **Article 5**  **1.The Commission, in cooperation with Member States and the relevant Union agencies, shall establish a Union health crisis and pandemic plan (‘the Union preparedness and response plan’) to promote effective and coordinated response to cross-border health threats at Union level.**  **[Population-based disease control]** | **Article 12**  **3.The Commission shall, in liaison with the Member States, ensure coordination and information exchange between the entities organizing any action, including, but not limited to joint procurement procedures, stockpiling and donation of medical countermeasures under different mechanisms established at Union level, in particular under:**    **[ Management of medical countermeasures, supplies and equipment]** | **Article 4**    **1.The Health Security Committee (’HSC’) is hereby established. It shall be composed of representatives of the Member States, in two working formations:**    **(a)a high-level working group to discuss topics of political importance and decisions referred to in point (d) of paragraph 3 and paragraph 7;**    **(b)technical working groups to discuss specific topics of technical nature.**    **[Crisis management]** | **Article 7**  **1. Member States shall by the end of November 2021 and every 2 years thereafter provide the Commission with a report on their preparedness and response planning and implementation at national level.**  **(b) elements of emergency preparedness, in particular:**  **(ii) capacities: including assessments of risks and capacities to determine priorities for emergency preparedness; surveillance and early warning, information management; access to diagnostic services during emergencies; basic and safe gender-sensitive health and emergency services; risk communications; research development and evaluations to inform and accelerate emergency preparedness;**    **[Communicate risk in an accurate, transparent and timely manner]** |
| **Article 13**  **1. The network for the epidemiological surveillance of the communicable diseases and of the related special health issues referred to in points (i) and (ii) of point (a) of Article 2(1) shall ensure a permanent communication between the Commission, the ECDC, and the competent authorities responsible at national level for epidemiological surveillance.**  **[Surveillance and epidemiologic monitoring ]** | **Article 5**  **4.The Union preparedness and response plan shall include interregional preparedness elements to establish coherent, multi-sectoral, cross-border public health measures, in particular considering capacities for testing, contact tracing, laboratories, and specialised treatment or intensive care across neighbouring regions. The plans shall include preparedness and response means to address the situation of those citizens with higher risks.**  **[Infection control and treatment guidance]** | **Article 25**  **1.The recognition of an emergency situation pursuant to Article 23 shall have the legal effect of enabling the introduction of:**  **(a)measures, which are applicable during the period of public health emergencies, related to medicinal products and medical devices provided for in Regulation (EU) …/… [OJ: Please insert the number of Regulation EMA [ISC/2020/12532]];**  **(b)mechanisms to monitor shortages of, develop, procure, manage and deploy medical countermeasures;**  **(c)activation of support from the ECDC as referred to in Regulation (EU) …/… [OJ: Please insert the number of Regulation ECDC [ISC/2020/12527]] to mobilise and deploy the EU Health Task Force.**    **[ Preventative services and Medical surge and Management of medical countermeasures, supplies and equipment ]** | **Article 4**    **2.The HSC shall have the following tasks:**    **(a)enabling of coordinated action by the Commission and the Member States for the implementation of this Regulation;**    **(b)coordination in liaison with the Commission of the preparedness and response planning of the Member States in accordance with Article 10;**  **[Crisis management]** | **Article 21**  **1. Following an alert notification pursuant to Article 19, on a request from the Commission or a Member State and on the basis of the available information, including the information referred to in Article 19 and the risk assessments referred to in Article 20, Member States shall coordinate within the HSC and in liaison with the Commission:**    **(b)risk and crisis communication, to be adapted to Member State needs and circumstances, aimed at providing consistent and coordinated information in the Union to the public and to healthcare professionals;**  **[ Communicate risk in an accurate, transparent and timely manner and Use dynamic listening, gather evaluation data, and manage rumors ]** |
| **Article 13**  **2. The epidemiological surveillance network shall aim to:**    **a.  Monitor trends in communicable disease over time and across MS and TC to assess situation, respond to rises above warning thresholds and facilitate appropriate evidence-based action**  **b.  Detect and monitor any multinational communicable disease outbreaks with respect to source, time population and place to provide rational for public health action**  **d.  Identify risk factors for disease transmission, population groups at risk and in need of targeted prevention measures**  **i.   Support the contact tracing measures of competent health authorities**  **[ Surveillance and epidemiologic monitoring and Risk Characterization and Incident recognition ]** | **Article 7**  **1. Member States shall by the end of November 2021 and every 2 years thereafter provide the Commission with a report on their preparedness and response planning and implementation at national level.**  **That report shall cover the following:**  **(a)identification of, and update on the status of the implementation of the capacity standards for preparedness and response planning as determined at national level for the health sector, as provided to the WHO in accordance with the IHR;**    **[ Enforcing laws and regulations]** |  | **Article 5**    **1.The Commission, in cooperation with Member States and the relevant Union agencies, shall establish a Union health crisis and pandemic plan (‘the Union preparedness and response plan’) to promote effective and coordinated response to cross-border health threats at Union level.**    **[Crisis management]** | **Article 24**  **1. For the purpose of the formal recognition of a public health emergency at Union level, the Commission shall establish an Advisory Committee on public health emergencies (‘Advisory Committee’) which, at the request of the Commission, shall advise the Commission by providing its views on:**    **c.  Advice on response including**    **i.   Formulation of response measures, including risk and crisis communication, to be addressed to all MS in line with the different stages of the threat in the Union**    **[ Communicate risk in an accurate, transparent and timely manner ]** |
| **Article 13**  **9. The Commission shall, by means of implementing acts, establish and update:**    **(b)case definitions concerning each communicable disease and related special health issue subject to epidemiological surveillance, in order to ensure the comparability and compatibility at Union level of the collected data;**    **(c)procedures for the operation of the epidemiological surveillance network as developed pursuant to Article 5 of Regulation (EU) …/… [OJ: Please insert the number of Regulation ECDC [ISC/2020/ 12527]].**  **[Epidemiologic Investigation]** | **Article 7**  **1. Member States shall by the end of November 2021 and every 2 years thereafter provide the Commission with a report on their preparedness and response planning and implementation at national level.**  **That report shall cover the following:**  **(b)elements of emergency preparedness, in particular:**    **(i)governance: including national policies and legislation that integrate emergency preparedness; plans for emergency preparedness, response and recovery; coordination mechanisms;**  **(ii) capacities: including assessments of risks and capacities to determine priorities for emergency preparedness; surveillance and early warning, information management; access to diagnostic services during emergencies; basic and safe gender-sensitive health and emergency services; risk communications; research development and evaluations to inform and accelerate emergency preparedness;**  **(iii) resources: including financial resources for emergency preparedness and contingency funding for response; logistics mechanisms and essential supplies for health; and dedicated, trained and equipped human resources for emergencies; and**  **(c) implementation of national response plans, including where relevant implementation at the regional and local levels, covering epidemic response; antimicrobial resistance, health care associated infection, and other specific issues.**  **[For infection control and treatment guidance and Population-based disease control]** |  | **Article 5**  **3. The Union preparedness and response plan shall, in particular,  include arrangements for governance, capacities and resources for:**    **a.  Timely cooperation between the Commission, MS and Union Agencies**    **b.  Secure exchange of information between Commission, Union Agencies and MS**    **f.   Health preparedness and response and intersectoral collaboration**    **[ Communication with other public health agencies at global, European, national and sub national level and Communication with emergency management, public safety and other sectors** |  |
| **Article 14**  **1. The ECDC shall ensure the further development of the digital platform through which data are managed and automatically exchanged, to establish integrated and interoperable surveillance systems enabling real-time surveillance where appropriate, for the purpose of supporting communicable disease prevention and control.**  **[ Surveillance and epidemiologic monitoring ]** | **Article 8**  **1. Every 3 years, the ECDC shall conduct audits in the Member States aimed at ascertaining the state of implementation of the national plans and their coherence with the Union plan. Such audits shall be implemented with the relevant Union agencies, aiming at the assessment of preparedness and response planning at national level with regard to the information referred to in Article 7(1).**  **[ Population-based disease control and enforcing laws and regulations]** |  | **Article 6**  **1 .When preparing national preparedness and response plans each Member State shall coordinate with the Commission in order to reach consistency with the Union preparedness and response plan, also inform without delay the Commission and the HSC of any substantial revision of the national plan.**  **[ Communication with other public health agencies at the global, European, national and subnational level ]** |  |
| **Article 15**  **1.  In the area of public health or for specific areas of public health relevant for the implementation of this Regulation or of the national plans referred to in Article 6, the Commission may, by means of implementing acts, designate EU reference laboratories to provide support to national reference laboratories to promote good practice and alignment by Member States on a voluntary basis on diagnostics, testing methods, use of certain tests for the uniform surveillance, notification and reporting of diseases by Member States.**  **[Laboratory Analysis]** | **Article 8**  **2. Member States shall present an action plan addressing the proposed recommendations of the audit and the corresponding corrective actions and milestones.**  **[ Population-based disease control and enforcing laws and regulations]** |  | **Article 10**  **1. The Commission and the Member States shall work together within the HSC to coordinate their efforts to develop, strengthen and maintain their capacities for the monitoring, early warning and assessment of, and response to serious cross-border threats to health.**  **The coordination shall, in particular, be aimed at:**  **(a)sharing best practice and experience in preparedness and response planning;**  **(b)promoting the interoperability of national preparedness planning and the intersectoral dimension of preparedness and response planning at Union level;**  **(c)supporting the implementation of capacity requirements for surveillance and response as referred to in the IHR;**  **(d)developing the preparedness plans referred to in Articles 5 and 6;**  **(e)monitoring progress, identifying gaps and actions to strengthen preparedness and response planning, including in the field of research, at national and at Union levels.**  **[Communication with other public health agencies at the global, European, national and subnational level and Communication with emergency management, public safety and other sectors ]** |  |
| **Article 15**  **2. The EU reference laboratories shall be responsible in particular for following tasks to coordinate the network of national reference laboratories:**    **a.  Reference diagnostics, including test protocols**  **b.  Reference material resources**  **c.  External quality assessments**  **d.  Scientific advice and technical assistance**  **e.  Collaboration and research**  **f.   Monitoring, alert and support in outbreak response**    **[Laboratory Analysis]** | **Article 9**    **2. The Commission may adopt recommendations on preparedness and response planning addressed to Member States based on the report referred to in paragraph 1.**    **[ Population-based disease control]** |  | **Article 11**    **1.The Commission may organise training activities for healthcare staff and public health staff in the Member States, including preparedness capacities under the International Health Regulations.**  **[ Communication with Healthcare providers ]** |  |
| **Article 17**  **1.Following an alert notified pursuant to Article 19 concerning a serious cross-border threat to health referred to in point (iii) of point (a) of Article 2(1) and in points (b), (c) or (d) of Article 2(1), Member States shall, in liaison with the Commission and on the basis of the available information from their monitoring systems, inform each other through the ‘Early Warning and Response System’ (‘EWRS’) and, if the urgency of the situation so requires, through the HSC about developments with regard to the threat concerned at national level.**  **[ Incident recognition and Surveillance and epidemiological monitoring ]** | **Article 18**  **1. The EWRS shall enable the Commission and the competent authorities responsible at national level to be in permanent communication for the purposes of preparedness, early warning and response, alerting, assessing public health risks and determining the measures that may be required to protect public health.**  **[ Population-based disease control ]** |  | **Article 13**    **1.The network for the epidemiological surveillance of the communicable diseases and of the related special health issues referred to in points (i) and (ii) of point (a) of Article 2(1) shall ensure a permanent communication between the Commission, the ECDC, and the competent authorities responsible at national level for epidemiological surveillance.**    **[ Communication with other public health agencies at the global, European, national and subnational level ]** |  |
| **Article 18**  **1. The EWRS shall enable the Commission and the competent authorities responsible at national level to be in permanent communication for the purposes of preparedness, early warning and response, alerting, assessing public health risks and determining the measures that may be required to protect public health.**    **[ Risk Characterization and Surveillance and epidemiologic monitoring ]** | **Article 20**  **1. Where an alert is notified pursuant to Article 19, the Commission shall, where necessary for the coordination of the response at Union level or upon request of the HSC referred to in Article 21 or on its own initiative, make promptly available to the national competent authorities and to the HSC, through the EWRS, a risk assessment of the potential severity of the threat to public health, including possible public health measures. That risk assessment shall be carried out by:**    **(a)the ECDC in accordance with Article 8a of Regulation (EU) …/… [OJ: Please insert the number of Regulation ECDC [ISC/2020/ 12527]] in the case of a threat referred to in points (i) and (ii) of point (a) of Article 2(1) including substances of human origin: blood, organs, tissues and cells potentially impacted by communicable diseases; or point (d) of Article 2(1); and/or**  **[Population-based disease control]** |  | **Article 17**    **1.Following an alert notified pursuant to Article 19 concerning a serious cross-border threat to health referred to in point (iii) of point (a) of Article 2(1) and in points (b), (c) or (d) of Article 2(1), Member States shall, in liaison with the Commission and on the basis of the available information from their monitoring systems, inform each other through the ‘Early Warning and Response System’ (‘EWRS’) and, if the urgency of the situation so requires, through the HSC about developments with regard to the threat concerned at national level.**    **[Communication with other public health agencies at the global, European, national and subnational level]** |  |
| **Article 19**    **1.National competent authorities or the Commission shall notify an alert in the EWRS where the emergence or development of a serious cross-border threat to health fulfils the following criteria:**    **(a) it is unusual or unexpected for the given place and time, or it causes or may cause significant morbidity or mortality in humans, or it grows rapidly or may grow rapidly in scale, or it exceeds or may exceed national response capacity; and**  **(b) it affects or may affect more than one Member State; and**  **(c) it requires or may require a coordinated response at Union level.**    **[Incident recognition ]** | **Article 21**  **1.Following an alert notification pursuant to Article 19, on a request from the Commission or a Member State and on the basis of the available information, including the information referred to in Article 19 and the risk assessments referred to in Article 20, Member States shall coordinate within the HSC and in liaison with the Commission:**  **(a) national responses, including research needs, to the serious cross-border threat to health, including where a public health emergency of international concern is declared in accordance with the IHR and falls within Article 2 of this Regulation**  **(c) adoption of opinions and guidance, including on specific response measures for the Member States for the prevention and control of a serious cross-border threat to health.**  **[ Population-based disease control]** |  | **Article 18**  **1.The EWRS shall enable the Commission and the competent authorities responsible at national level to be in permanent communication for the purposes of preparedness, early warning and response, alerting, assessing public health risks and determining the measures that may be required to protect public health.**  **[Communication with other public health agencies at the global, European, national and subnational level And Crisis Management ]** |  |
| **Article 20**  **1. Where an alert is notified pursuant to Article 19, the Commission shall, where necessary for the coordination of the response at Union level or upon request of the HSC referred to in Article 21 or on its own initiative, make promptly available to the national competent authorities and to the HSC, through the EWRS, a risk assessment of the potential severity of the threat to public health, including possible public health measures. That risk assessment shall be carried out by:**    **(a)the ECDC in accordance with Article 8a of Regulation (EU) …/… [OJ: Please insert the number of Regulation ECDC [ISC/2020/ 12527]] in the case of a threat referred to in points (i) and (ii) of point (a) of Article 2(1) including substances of human origin: blood, organs, tissues and cells potentially impacted by communicable diseases; or point (d) of Article 2(1); and/or**  **[Risk characterization]** | **Article 22**  **1.The Commission may complement the action of the Member States through the adoption of recommendations on common temporary public health measures for Member States.**  **[ Population-based disease control ]** |  | **Article 19**    **1.National competent authorities or the Commission shall notify an alert in the EWRS where the emergence or development of a serious cross-border threat to health fulfils the following criteria:**  **[ Communication with other public health agencies at the global, European, national and subnational level ]** |  |
| **Article 20 paragraph 3**  **Where the risk assessment needed is totally or partially outside the mandates of the agencies referred to in paragraph 1 and is considered necessary for the coordination of the response at Union level, the Commission shall, upon request of the HSC or its own initative, provide an ad hoc risk assessment**  **[Risk characterization]** | **Article 22**  **2. The recommendation for measures adopted under paragraph 1 shall:**    **(a) be based on in particular recommendations of the ECDC in particular, other relevant agencies or bodies, or the Advisory Committee referred to in Article 24;**    **(b) respect the responsibilities of the Member States for the definition of their health policy and for the organisation and delivery of health services and medical care;**    **(c) be proportionate to the public health risks related to the threat in question, avoiding in particular any unnecessary restriction to the free movement of persons, of goods and of services.**  **[Enforcement of laws and regulations ]** |  | **Article 21**  **1.Following an alert notification pursuant to Article 19, on a request from the Commission or a Member State and on the basis of the available information, including the information referred to in Article 19 and the risk assessments referred to in Article 20, Member States shall coordinate within the HSC and in liaison with the Commission:**    **(a)national responses, including research needs, to the serious cross-border threat to health, including where a public health emergency of international concern is declared in accordance with the IHR and falls within Article 2 of this Regulation;**  **(b)risk and crisis communication, to be adapted to Member State needs and circumstances, aimed at providing consistent and coordinated information in the Union to the public and to healthcare professionals;**  **(c)adoption of opinions and guidance, including on specific response measures for the Member States for the prevention and control of a serious cross-border threat to health.**  **[Crisis management and Communication with other public health agencies at the global, European, national, and subnational level and Communication with healthcare providers ]** |  |
| **Article 23**    **1.The Commission may, based on the expert opinion of the Advisory Committee referred to in Article 24, formally recognise a public health emergency at Union level; including pandemic situations where the serious cross-border threat to health in question endangers public health at the Union level.**  **[Incident recognition]** | **Article 24**  **1. For the purpose of the formal recognition of a public health emergency at Union level, the Commission shall establish an Advisory Committee on public health emergencies (‘Advisory Committee’) which, at the request of the Commission, shall advise the Commission by providing its views on:**  **(c)advice on response including:**  **(ii) identification and mitigation of significant gaps, inconsistencies or inadequacies in measures taken or to be taken to contain and manage the specific threat and overcome its impact, including in clinical management and treatment, non-pharmaceutical countermeasures and public health research needs;**  **(iv) subsequently, recommendation of policy measures for addressing and mitigating long-term consequences of the specific threat.**  **[ Population-based disease control and Infection control and treatment guidance]** |  | **Article 21**    **2.Where a Member State intends to adopt public health measures to combat a serious cross-border threat to health, it shall, before adopting those measures, inform and consult the other Member States and the Commission on the nature, purpose and scope of the measures, unless the need to protect public health is so urgent that the immediate adoption of the measures is necessary.**    **[ Crisis management and Communication with other public health agencies at the global, European, national, and subnational level ]** |  |
| **Article 24**    **1.For the purpose of the formal recognition of a public health emergency at Union level, the Commission shall establish an Advisory Committee on public health emergencies (‘Advisory Committee’) which, at the request of the Commission, shall advise the Commission by providing its views on:**    **(a)whether a threat constitutes a public health emergency at Union level;**    **(b)the termination of a public health emergency at Union level; and**  **[ Incident recognition ]** |  |  |  |  |

# Proposal for a Regulation amending Regulation No 851/2004 establishing a ECDC

*Supplementary Table 2.  Analysis of Proposal for a Regulation amending Regulation (EC) No 851/2004 establishing a European Centre for disease prevention and control and classification of provisions into Public Health Emergency capabilities according to Stoto et al (2017) PHE logic model.*

| ***Detection and Assessment*** | ***Policy Development, Adaptation and Implementation*** | ***Healthcare Services*** | ***Coordination and Communication*** | ***Emergency Risk Communication*** |
| --- | --- | --- | --- | --- |
| **Article 3 Paragraph 1**  Mission of the Centre shall be to identify assess and report on current and emerging threats to human health from communicable diseases, and provide recommendations for response at Union and national levels as well as at regional level if necessary    In the case of other outbreaks of illnesses of unknown origin that may spread within or to the Union, the Centre shall act on its own initiative until the source of the outbreak is known    [**Risk Characterization and Surveillance and epidemiologic monitoring and Incident recognition and Epidemiological investigation ]** | **Article 3 Paragraph 2**  The Centre shall, within its financial capacity and mandate, perform the following tasks:    b.      Provide analyses, scientific advice, opinions and support for actions by the Union and MS on SCBTH including risk assessments, analysis of epidemiological info, epidemiological modelling, anticipation and forecast, recommendations for actions to prevent and control communicable disease threats and other special health issues, contribution to defining research priorities, and scientific and technical assistance including training and other activities within its mandate  d.      Exchange information, expertise and best practice  [ **Population-Based Disease Control** ] | **Article 3 Paragraph 2**  The Centre shall, within its financial capacity and mandate, perform the following tasks:    h.  Support epidemic and outbreak response in Member States, and in third countries, in complementarity with other Union emergency response instruments, in particular EU partner countries  [ **Medical Surge** ] | **Article 3 paragraph 2**  The Centre shall, within its financial capacity and mandate, perform the following tasks:    c. Coordinate the European networking of bodies operating in the fields within the Centre’s mission, including networks arising from public health activities supported by the Commission and operating the dedicated networks    [**Communication with other public health agencies at the global, European, national, and subnational level and Communication with health care providers ]** | **Article 3 paragraph 2**  The Centre shall, within its financial capacity and mandate, perform the following tasks:    j.       Provide upon request of the Commission or the HSC, evidence-based communication messages to the public on communicable diseases, on the threats to health posed by them and on the relevant prevention and control measures    [ **Communicate risk in an accurate, transparent and timely manner** ] |
| **Article 3 Paragraph 2**  The Centre shall, within its financial capacity and mandate, perform the following tasks:    a.      Search for, collect, collate, evaluate and disseminate relevant scientific and technical data and information, considering the latest technologies  b.      Provide analyses, scientific advice, opinions and support for actions by the Union and MS on SCBTH including risk assessments, analysis of epidemiological info, epidemiological modelling, anticipation and forecast, recommendations for actions to prevent and control communicable disease threats and other special health issues, contribution to defining research priorities, and scientific and technical assistance including training and other activities within its mandate  [ **Risk Characterization and Surveillance and epidemiologic monitoring ]** | **Article 3 paragraph 2**  The Centre shall, within its financial capacity and mandate, perform the following tasks:    g. Provide upon request of the Commission or HSC, or its own initative, guidelines for treatment and case management of communicable diseases and other special health issues relevant for public health in cooperation with relevant societies    [**Infection control and treatment guidance** ] | **Article 5a para 4**    Centre coordinate independent post marketing vaccines effectiveness and safety monitoring studies collecting new information and or using the relevant data collected by competent bodies. That work shall be conducted jointly with the EMA and through a new vaccine monitoring platform.    [**Preventative services** | **Article 3 paragraph 2**  The Centre shall, within its financial capacity and mandate, perform the following tasks:    f.       Monitor health systems capacity relevant to the management of communicable disease threats and other special health issues    [ **Crisis Management** ] |  |
| **Article 5 para 1**    Centre shall ensure operation of network for epidemiological surveillance of diseases    [ **Surveillance and epidemiological monitoring** ] |  | **Article 11a**    **P1** The Centre shall establish capacity to mobilise and deploy the EU Health Task Force including the Centre’s staff and experts from Member States and fellowship programmes, to assist local response to outbreaks of communicable diseases in Member States and in third countries.    **P3** The Centre shall ensure that the EU Health Task Force are coordinated and complementary to the capacities integrating the European Medical Corps and other relevant capacities under the Union Civil Protection Mechanism.    **P4** The Centre shall develop with the Commission a framework for the mobilisation of the EU Health Task Force, in view of action under Decision No 1313/2013/EU*  The Centre shall provide contributions of Union field response experts in international response teams mobilised by the WHO Health Emergencies Programme mechanism and the Global Outbreak Alert and Response Network (GOARN) and in accordance with appropriate working arrangements established with the Commission.      **P5** The Centre shall facilitate the development of field response capabilities and crisis management expertise among the Centre’s staff and experts from EU and EEA countries, EU candidate countries and potential candidates, as well as European Neighbourhood Policy and EU partner countries, upon request of the Commission  [ **Medical Surge**] | **Article 4**    Member States shall:    a.      Communicate to Centre in timely manner and according to agreed …. On surveillance of communicable diseases and available scientific and tech data and information relevant to Centre’s mission including info relevant to the Centre’s mission and health systems capacities to detect, prevent, respond to and recover from outbreaks of communicable disease  b.      Notify centre of any SCBTH as soon as detected through the EWRS and promptly communicate response measures taken as well as any relevant information that may be useful for coordinating the response    [**Crisis Management and Communication with other public health agencies at the global, European, national, and subnational level** |  |
| **Article 5 Para 4**    The Centre, through the operation of the network for the epidemiological surveillance, shall:    (a)monitor and report on trends in communicable diseases over time and across, to assess the present situation and facilitate appropriate evidence-based action, including through the identification of specifications for harmonised data collection from member states    (b)detect, monitor and report on serious cross-border threats to health in the case of a threat    (c)contribute to the evaluation and monitoring of communicable disease prevention and control programmes in order to provide the evidence for recommendations to strengthen and improve these programmes at the national and Union levels;    (d)monitor and assess health systems’ capacity for diagnosis, prevention and treatment of specific communicable diseases as well as patients’ safety;    (e)identify population groups at risk and in need of targeted prevention and response measures, and ensure that those measures are accessible for persons with disabilities;    (f)contribute to the assessment of the burden of communicable diseases on the population using data, such as disease prevalence, complications, hospitalisation and mortality, and ensure that this data is disaggregated on age, gender and disability;    (g)carry out epidemiological modelling, anticipation and scenario development for response and coordinate such efforts with a view to exchange best practices and improve modelling capacity across the Union; and    (h)identify risk factors for disease transmission, groups most at risk, including the correlation of disease incidence and severity with societal and environmental factors, and research priorities and needs.  [**Risk Characterization AND Epidemiological Investigation AND Surveillance and Epidemiological Monitoring AND Incident Recognition]** |  |  | **Article 5 Para 3**    Centre shall support work of HSC, Council and other Union structures for coordinating responses to SCBTH in its mandate    [**Crisis management**] |  |
| **Article 5 para 6**  Centre shall ensure operation of network of EU reference labs for diagnosis, detection identification and characterization of infectious agents    [**Laboratory services**] |  |  |  |  |
|  | **Article 6 para  1a:**    The centre shall provide concrete analyses and recommendations for actions to prevent and control communicable disease threats upon request of the Commission    [**Population based disease control** ] |  |  |  |
| **Article 8a**    P1 centre shall provide timely rapid risk asssessments…    P2 Risk assessment shall include general and targeted recommendations for response as a basis for coordination in the HSC  [ **Risk Characterization]** | **Article 8b**    P1 Centre shall support response coordination in HSC in particular by providing recommendations for response measures for  a.      Ntl responses to SCBTH  b.      Adoption of guidance for MS for prevention and control of a serious cross border threat to health  [ **Population based disease control** ] |  |  |  |
| **Article 9 paragraph 2**    Centre may be requested by Commission, Member State, Health Security Committee, international organisation to provide scientific or tech assistance in any field within the scope of its mission …. Include aiding Commission and Member States to develop technical guidelines on good practice and on protective measures to be taken in response to human health threats, providing expert assistance mobilizing and coordinating investigation teams. The Centre shall provide scientific and technical expertise and assistance within its financial capacity and mandate, and in accordance with appropriate working arrangements established with commission.  [ **Epidemiological investigation** ] |  |  |  |  |

# Regulation (EU) 2022/123 on reinforced role for the EMA in crisis preparedness and management for medicinal products and medical devices

*Supplementary Table 3.  Analysis of Newly adopted Regulation (EU) 2022/123 on reinforced role for the EMA in crisis preparedness and management for medicinal products and medical devices*

| **Detection and Assessment** | **Policy Development, Adaptation and Implementation** | **Healthcare Services** | **Coordination and Communication** | **Emergency Risk Communication** |
| --- | --- | --- | --- | --- |
| **Article 4**    1.   The Agency, in collaboration with Member States, shall continuously monitor any event that is likely to lead to a public health emergency or major event. As necessary, the Agency shall cooperate with the European Centre for Disease Prevention and Control (‘ECDC’) and, where relevant, other Union agencies.    [ **Surveillance and epidemiological monitoring** ] | **Article 8**    3.   As part of the reporting referred to in paragraphs 1 and 2, the MSSG may provide recommendations on measures that the Commission, Member States, marketing authorisation holders and other entities, including representatives of healthcare professionals and of patients, could take to prevent or mitigate actual or potential shortages of medicinal products.    Member States may request the MSSG to provide recommendations on measures referred to in the first subparagraph.    For the purposes of the second subparagraph, the MSSG shall liaise, as relevant, with the HSC and, in the case of a public health emergency, with any other relevant advisory committee on public health emergencies established pursuant to Union law.    [ **Policy Development – Medicine shortages** ] | **Article 1**    Within the European Medicines Agency (the ‘Agency’),this Regulation provides for a framework for and the means of:    (a)    preparing for, preventing, coordinating and managing the impact of public health emergencies on medicinal products and on medical devices and the impact of major events on medicinal products and on medical devices at Union level;    (b)    monitoring, preventing, and reporting on shortages of medicinal products and on shortages of medical devices;    (c)    setting up an interoperable information technology (IT) platform at Union level to monitor and report on shortages of medicinal products;    [ **Management of Medical countermeasures** ] | **Article 8**    5.   Where requested by the Commission, the MSSG may coordinate measures taken by the national competent authorities, the marketing authorisation holders and other entities, including representatives of healthcare professionals and of patients, as relevant, to prevent or mitigate actual or potential shortages of medicinal products in the context of a public health emergency or major event.    [ **Crisis management** ] | **Article 6**    6.   The Agency shall establish within its web portal a publicly accessible webpage that provides information on actual shortages of medicinal products included in the critical medicines lists in cases in which the Agency has assessed the shortage and has provided recommendations to healthcare professionals and patients. The webpage shall provide at least the following information:    (a)    the name and common name of the medicinal product on the critical medicines lists;    (b)    the therapeutic indications for the medicinal product on the critical medicines lists;    (c)    the reason for the shortage of the medicinal product on the critical medicines lists;    (d)    the start and end dates of the shortage of the medicinal product on the critical medicines lists;    (e)    the Member States affected by the shortage of the medicinal product on the critical medicines lists;    (f)    other relevant information for healthcare professionals and patients, including information on whether alternative medicinal products are available.    The webpage referred to in the first subparagraph shall also provide references to national registries on shortages of medicinal products.    [ **Communication with Health care providers** ] |
| **Article 23**    1.   During a public health emergency the MDSSG shall monitor the supply of and demand for medical devices included on the public health emergency critical devices list, with a view to identifying any actual or potential shortages of those medical devices. The MDSSG shall conduct such monitoring using the public health emergency critical devices list and the information and data provided in accordance with Articles 26 and 27.    [ **Surveillance and epidemiological monitoring** ] | **Article 8**    4.   The MSSG, on its own initiative or at the request of the Commission or a Member State, may provide recommendations on measures that the Commission, Member States, marketing authorisation holders, representatives of healthcare professionals and other entities could take to ensure preparedness for dealing with actual or potential shortages of medicinal products caused by public health emergencies or major events.    **[ Policy development – medicine shortage ]** | **Article 3**    4.   The MSSG, in coordination with the national competent authorities for medicinal products, shall facilitate appropriate communication with marketing authorisation holders or their representatives, manufacturers, other relevant actors of the supply chain for medicinal products, and representatives of healthcare professionals, of patients and consumers, with a view to receiving relevant information on actual or potential shortages of medicinal products considered to be critical during a public health emergency or a major event as provided for in Article 6.    [ **Management of Medical countermeasures** ] | **Article 15**    2.   During public health emergencies, the ETF shall undertake the following tasks:      (f)    cooperating with national competent authorities, Union bodies and agencies, the World Health Organization, third countries, and international scientific organisations, on scientific and technical issues that relate to the public health emergency and to medicinal products which have the potential to address public health emergencies, as necessary.    [**Communication with other public health agencies at the global, European, national and subnational levels**] | **Article 14**    1.   The Agency shall provide information to the public and interest groups with regard to the work of the MSSG in a timely manner and shall respond to disinformation targeting the work of the MSSG as appropriate, via a dedicated webpage on its web portal and other appropriate means, in cooperation with national competent authorities.    [ **Use dynamic listening, gather evaluation data and manage rumors**] |
| **Article 24**    2.   Where requested by the Commission, Member States or one or more single point of contact referred to in Article 25(2), point (a), the MDSSG shall provide aggregated data and demand forecasts to support its findings and conclusions.    For the purposes of the first subparagraph, the MDSSG shall liaise with the ECDC to obtain epidemiological data to help forecast medical device needs, and with the MSSG where medical devices included on the public health emergency critical devices list are used jointly with a medicinal product.    The findings and conclusions of the MDSSG referred to in the first subparagraph may be made available to other actors in the medical device sector, where appropriate, in accordance with competition law, with a view to better preventing or mitigating or actual or potential shortages.    [ **Surveillance and epidemiological monitoring** ] | **Article 12**    The Commission shall take into account the information from and recommendations of the MSSG referred to in Article 8(1) and (2) and in Article 8(3) and (4), respectively, and shall: […]    (b)    facilitate the coordination between marketing authorisation holders and other relevant entities to address demand surges, where necessary;    (c)    consider the need for guidelines and recommendations to be addressed to Member States, marketing authorisation holders, and other entities, including relevant entities from the supply chain for medicinal products, where relevant;    [ **Policy development – medicine shortages** ] | **Article 5**    1.   Following the recognition of a public health emergency or the recognition of a major event in accordance with Article 4(3), the MSSG shall evaluate information related to the public health emergency or the major event and consider the need for urgent and coordinated action with regard to the quality, safety and efficacy of the medicinal products concerned.    [ **Management of Medical countermeasures** ] | **Article 24**    5.   Where requested by the Commission, the MDSSG may coordinate measures taken by the national competent authorities for medical devices, manufacturers of medical devices, notified bodies, and other entities, as relevant, to prevent or mitigate actual or potential shortages of medical devices in the context of a public health emergency or major event.    [ **Crisis management** ] | **Article 19**    The Agency shall provide information to the public and relevant interest groups with regard to the work of the ETF in a timely manner and shall respond to disinformation targeting the work of the ETF, as appropriate, via a dedicated webpage on its web portal and other appropriate means, in cooperation with national competent authorities.    [ **Use dynamic listening, gather evaluation data and manage rumors**] |
|  | **Article 15**    2.   During public health emergencies, the ETF shall undertake the following tasks:    (b)    providing advice on the main aspects of clinical trial protocols, and providing advice to developers on clinical trials for medicinal products intended to treat, prevent or diagnose the disease causing the public health emergency, in accordance with Article 16 of this Regulation without prejudice to the tasks of the Member States as regards the assessment of submitted clinical trial applications to be conducted within their territories in accordance with Regulation (EU) No 536/2014;    [ **Policy development – non specific – developing and approving  medicines during times of crisis** ] | **Article 6**    1.   Without prejudice to paragraph 2, the MSSG shall establish a list with the main therapeutic groups of medicinal products that are necessary for emergency care, surgery and intensive care, in order to inform the preparation of the critical medicines lists as referred to in paragraphs 2 and 3 to be used to respond to a public health emergency or major event. The list shall be established by 2 August 2022 and updated annually and whenever necessary.    [ **Preventive Services**] |  | **Article 29**    1.   The Agency shall provide information to the public and relevant interest groups with regard to the work of the MDSSG in a timely manner and shall respond to disinformation targeting the work of the MDSSG, as appropriate, via a dedicated webpage on its web portal and other appropriate means, in cooperation with national competent authorities.    [ **Use dynamic listening, gather evaluation data and manage rumours** ] |
|  | **Article 20**    In preparation for and to support the work of the ETF during public health emergencies, the Agency shall: […]      (b)    coordinate independent monitoring studies on the use, effectiveness and safety of medicinal products intended to treat, prevent or diagnose diseases related to the public health emergency, using relevant data, including, where relevant, data held by public authorities;    [ **Infection control and treatment guidance** ] | **Article 7**    Following the recognition of a public health emergency or the recognition of a major event in accordance with Article 4(3), the MSSG shall monitor the supply of and demand for medicinal products included on the critical medicines lists, with a view to identifying any actual or potential shortages of those medicinal products. The MSSG shall conduct such monitoring using the critical medicines lists and the information and data provided, in accordance with Articles 10 and 11, and available through the ESMP, once it is fully functional.    [ **Management of Medical countermeasures** ] |  |  |
|  | **Article 24**    3.   As part of the reporting referred to in paragraphs 1 and 2, the MDSSG may provide recommendations on measures that the Commission, Member States, medical device manufacturers, notified bodies and other entities could take to prevent or mitigate actual or potential shortages of medical devices.    [ **Policy development – medicine / medical device shortage** ] | **Article 12**  The Commission shall take into account the information from and recommendations of the MSSG referred to in Article 8(1) and (2) and in Article 8(3) and (4), respectively, and shall:      (f) consider the need for medical countermeasures in accordance with Decision No 1082/2013/EU and other applicable Union law;    [ **Management of Medical countermeasures** ] |  |  |
|  | **Article 28**    The Commission shall take into account the information from and recommendations of the MDSSG and shall:      (b)    consider the need for guidelines and recommendations to be addressed to Member States, manufacturers of medical devices, notified bodies, and other entities, where relevant;    [ **Policy development – shortages of medicines and medical devices**] | **Article 13**    1.   The Agency shall set up, maintain, and manage an IT platform to be known as the European shortages monitoring platform (‘ESMP’), which shall be linked to the database referred to in Article 57(1), point (l), of Regulation (EC) No 726/2004.    The ESMP shall be used to facilitate the collection of information on shortages of, supply of, and demand for medicinal products, including information on whether the medicinal product is placed or ceases to be placed on the market in a Member State.    [ **Management of Medical countermeasures** ] |  |  |
|  | **Article 28**    The Commission shall take into account the information from and recommendations of the MDSSG and shall:    (c)    request the MDSSG to provide recommendations or coordinate measures provided for in Article 24(3), (4) and (5);    [ **Policy development – shortages of medicines and medical devices**] | **Article 13**    2.   The information collected through the ESMP shall be used to monitor, prevent, and manage:    (a)    actual or potential shortages of medicinal products on the critical medicines lists during public health emergencies and major events; and    (b)    actual or potential shortages of medicinal products that are likely to lead to a public health emergency or a major event in accordance with Article 4(2).    [ **Management of Medical countermeasures** ] |  |  |
|  |  | **Article 15**    2.   During public health emergencies, the ETF shall undertake the following tasks:    (a)    in liaison with the scientific committees, working parties, and scientific advisory groups of the Agency, providing scientific advice and reviewing the available scientific data on medicinal products that have the potential to address the public health emergency, including requesting data from developers and engaging with them in preliminary discussions;    [ **Preventative services** ] |  |  |
|  |  | **Article 16**    1.   During a public health emergency, the ETF shall provide advice on the main aspects of clinical trials and clinical trial protocols submitted or intended to be submitted in a clinical trial application by developers as part of an accelerated scientific advice process, without prejudice of the responsibility of the Member State or States concerned under Regulation (EU) No 536/2014.    [ **Preventative services** ] |  |  |
|  |  | **Article 18**    1.   Following the recognition of a public health emergency, the ETF shall undertake a review of the available scientific data on medicinal products which have the potential to be used to address the public health emergency. That review shall be updated whenever needed during the public health emergency, including where the ETF and the CHMP agree on the preparation of the assessment of a marketing authorisation application.    [ **Management of medical countermeasures** ] |  |  |
|  |  | **Article 21**    1.   The Executive Steering Group on Shortages of Medical Devices (the ‘Medical Device Shortages Steering Group – MDSSG’) is hereby established within the Agency.    [ **Management of medical countermeasures** ] |  |  |
|  |  | **Article 22**    1.   Immediately following the recognition of a public health emergency, the MDSSG shall consult the working party referred to in Article 21(5). Immediately following that consultation, the MDSSG shall adopt a list of categories of critical medical devices which it considers to be critical during the public health emergency (‘public health emergency critical devices list’).    [ **Management of medical countermeasures** ] |  |  |
|  |  | **Article 28**    The Commission shall take into account the information from and recommendations of the MDSSG and shall:    (a)    take all necessary action within the limits of the powers conferred on the Commission, with a view to mitigating actual or potential shortages of medical devices included on the public health emergency critical devices list, including, where necessary, granting temporary exemptions at Union level pursuant to Article 59(3) of Regulation (EU) 2017/745 or Article 54(3) of Regulation (EU) 2017/746, while respecting the conditions set out in those Articles and seeking to ensure both patient and product safety;    **[ Management of medical countermeasures ]** |  |  |
|  |  | **Article 28**    The Commission shall take into account the information from and recommendations of the MDSSG and shall:    (d)    consider the need for medical countermeasures in accordance with Decision No 1082/2013/EU and other applicable Union law;    **[ Management of medical countermeasures ]** |  |  |
|  |  | **Article 5**    2.   The MSSG shall provide recommendations to the Commission and Member States on any appropriate action that it believes needs to be taken at Union level on the medicinal products concerned in accordance with Directive 2001/83/EC or Regulation (EC) No 726/2004.    **[ Management of medical countermeasures ]** |  |  |

# Proposal for Council Regulation on a framework of measures for ensuring the supply of crisis-relevant medical countermeasures in the event of a public health emergency at Union level

*Supplementary Table 4.  Analysis of Proposal for Council Regulation on a framework of measures for ensuring the supply of crisis-relevant medical countermeasures in the event of a public health emergency at Union level*

| **Detection and Assessment** | **Policy Development, Adaptation and Implementation** | **Healthcare Services** | **Coordination and Communication** | **Emergency Risk Communication** |
| --- | --- | --- | --- | --- |
| **Article 3**   1. In the event of recognition of a public health emergency the Council, upon the proposal of the Commission, may adopt a regulation activating the emergency framework where that is appropriate to the economic situation. **[Incident Recognition]** |  | **Article 5**  1. Where this measure is activated, the Health Crisis Board shall be set up. It shall ensure coordination of action by the Council, the Commission, the relevant Union agencies and bodies, and Member States to ensure the supply of and access to medical countermeasures.  The coordination shall, in particular, be aimed at supporting the Commission in the preparation of measures to be taken pursuant to Articles 6 to 11 and 13. [**Management of medical countermeasures, supplies & equipment**] | **Article 6**  4. Where a Member State intends to adopt measures for the procurement, purchase or manufacturing of crisis-relevant medical countermeasures or raw materials, it shall inform and consult the Health Crisis Board.  [**Crisis management**] |  |
|  |  | **Article 5**  3. The Health Crisis Board shall ensure coordination and information exchange with the structures established under:  (a) Regulation (EU) …/… [the EMA Regulation] during the period of the public health emergency, related to medicinal products and medical devices;  (b) Regulation (EU) …/… [the SCBTH Regulation];  (c) Decision No 1313/2013/EU and in particular the Emergency Response Coordination Centre for the purpose of bridging operational gaps in accessing medical countermeasures and raw materials and ensuring, where necessary, corresponding on-site monitoring and coordination tasks.    [ **Crisis management OR/ Communication with other PH agencies**] | **Article 6**  7. The Commission shall provide information on the results of the monitoring of crisis- relevant medical countermeasures and raw materials to the European Parliament and the Council through the Integrated Political Crisis Response provided for in Council Implementing Decision (EU) 2018/199331.  The Commission shall make available to the European Parliament and the Council through the Integrated Political Crisis Response, modelling and forecasts regarding the needs for crisis-relevant medical countermeasures and raw materials with the support of relevant Union agencies, where appropriate.    **[Crisis management]** |  |
|  |  | **Article 6**  1. Where this measure is activated, the Commission shall, after seeking the advice of the Health Crisis Board, draw up and regularly update a list of crisis-relevant medical countermeasures and raw materials, as well as a template for monitoring their supply and demand, including production capacity, stockpiles, possible critical aspects or the risk of disruption in the supply chains and purchasing agreements.    **[ Management of medical countermeasures]** | **Article 11**  2. In particular, the measures referred to in paragraph 1 shall include;    (d) facilitating the collaboration of relevant companies in a joint industry effort to ensure the availability and supply of crisis-relevant medical countermeasures;  **[Crisis management]** |  |
|  |  | **Article 6**  2. The list referred to in paragraph 1 shall include a shortlist of specific crisis-relevant medical countermeasures and raw materials for the preparation of measures to be taken in accordance with this article and Articles 7 to 11 and 13, taking into account the information obtained pursuant to:  (a) Regulation (EU) …/… [the EMA Regulation] and in particular Articles XX [Article numbers to be confirmed after adoption] thereof, concerning the monitoring and mitigating shortages of critical medicinal products, medical devices and in vitro diagnostic medical devices;  (b) Regulation (EU) …/… [the ECDC Regulation], and in particular Article 3, point (e), thereof, concerning available indicators of Member States’ capacity regarding health services necessary to the management and response to communicable disease threats.    **[Management of Medical Countermeasures]** |  |  |
|  |  | **Article 6**  5. Upon request of the Commission, EMA shall provide it with information with regard to monitoring of medicinal products, medical devices and in vitro diagnostic medical devices, including their demand and supply, in accordance with Articles XX [Article numbers to be confirmed after adoption] of Regulation (EU) …/… [the EMA Regulation].    **[Management of Medical countermeasures]** |  |  |
|  |  | **Article 6**  6. The Commission shall gather information through a secured IT system and monitor all relevant information concerning the supply and demand of crisis-relevant medical countermeasures and raw materials within and outside the Union. The interoperability of the IT system with the electronic monitoring and reporting systems developed by EMA pursuant to Article 9, point (c), [Article numbers to be confirmed after adoption], of Regulation (EU) …/… [the EMA Regulation] shall be ensured by the Commission when necessary.    **[Management of Medical countermeasures]** |  |  |
|  |  | **Article 7**  1. Where this measure is activated, a negotiating mandate shall be established by the Commission on behalf of Member States that wish to be represented by the Commission (‘participating Member States’) to act as a central purchasing body for crisis-relevant medical countermeasures through the activation of existing contracts or the negotiation of new contracts using all available instruments, such as Article 4 of Regulation (EU) 2016/369; the joint procurement procedure referred to in Article 12 of Regulation (EU) …/… [the SCBTH Regulation], or European Innovation Partnerships.    **[Management of Medical Countermeasures]** |  |  |
|  |  | **Article 7**  3. In line with the negotiating mandate given to it, the Commission may have the ability and responsibility, on behalf of all participating Member States, to enter into purchase agreements with economic operators, including individual producers of crisis-relevant medical countermeasures, concerning the purchase of such countermeasures or concerning the advance financing of the production or the development of such countermeasures in exchange for a right to the result.  In order to prepare the fulfilment of such tasks, representatives of the Commission or experts nominated by the Commission may carry out on-site visits at the locations of production facilities of crisis-relevant medical countermeasures.    **[Management of Medical Countermeasures]** |  |  |
|  |  | **Article 7**  4. The Commission shall have the ability and responsibility to activate the EU-FAB facilities in order to make available reserved surge manufacturing capacities to ensure the delivery of crisis-relevant medical countermeasures and raw materials, corresponding to the agreed quantities and in accordance with the timing of the EU- FAB contracts. Specific procurement procedures for these agreed quantities of crisis- relevant medical countermeasures shall be conducted.    **[Management of Medical Countermeasures]** |  |  |
|  |  | **Article 7**  6. The Commission shall carry out the procurement procedures and conclude the resulting agreements with economic operators on behalf of the participating Member States. The Commission shall invite Member States participating in the Health Crisis Board set up under Article 5 to nominate representatives to take part in the preparation of the procurement procedures as well as the negotiation of the purchasing agreements. The deployment and use of the crisis-relevant medical countermeasures shall remain the responsibility of the participating Member States.  [**Management of Medical Countermeasures**] |  |  |
|  |  | **Article 8**  1. Where this measure is activated, the Commission and the Member States shall activate the emergency research and innovation aspects of the Union Preparedness and Response Plan referred to in Regulation (EU) …/… [the SCBTH Regulation].  **[Management of Medical Countermeasures]** |  |  |
|  |  | **Article 8**  4. The participation and contribution of the Union in the emergency research and innovation aspects of the Union Preparedness and Response Plan with the Member States shall be in accordance with the rules and procedures of the various Multiannual Financial Framework programmes.  **[Management of Medical Countermeasures]** |  |  |
|  |  | **Article 9**  1. Where this measure is activated, the Commission may, after consulting the Health Crisis Board, establish an inventory, and for this purpose request the producers of crisis-relevant medical countermeasures to inform the Commission within 5 days about the actual total production capacity and possible existing stocks of the crisis- relevant medical countermeasures and components thereof in its Union production facilities and third country facilities which it operates or contracts or purchases supply from, and to transmit to the Commission a schedule of the expected production output for the following 3 months for each Union production facility.  **[Management of Medical Countermeasures]** |  |  |
|  |  | **Article 9**  2. Upon request of the Commission, each producer of crisis-relevant medical countermeasures shall inform the Commission within a maximum of 5 days about any Union crisis-relevant medical countermeasures production facility it operates, including information on its production capacity as regards crisis-relevant medical countermeasures via regular updates. For medicinal products, this information shall comprise facilities related to both finished products as well as active pharmaceutical ingredients.  **[Management of Medical Countermeasures]** |  |  |
|  |  | **Article 10**  Where this measure is activated, the Commission shall extend the inventory provided for in Article 9 to crisis-relevant relevant raw materials, consumables, devices, equipment and infrastructure, if it considers that there is a risk of a shortage in supply of crisis-relevant raw materials, consumables, devices, equipment or any problems with infrastructure.  **[Management of Medical Countermeasures]** |  |  |
|  |  | **Article 11**  1. Where this measure is activated, the Commission shall, when it considers that there is a risk of a shortage of crisis-relevant raw materials, consumables, devices, equipment and infrastructure, implement together with the relevant Member States specific measures to ensure the efficient re-organisation of supply chains and production lines and utilise existing stocks to increase the availability and supply of crisis-relevant medical countermeasures, as quickly as possible.  **[Management of Medical Countermeasures]** |  |  |
|  |  | **Article 11**  2. In particular, the measures referred to in paragraph 1 shall include;    (a) facilitating the expansion or repurposing of existing or the establishment of new production capacities for crisis-relevant medical countermeasures;  [**Management of Medical Countermeasures**] |  |  |
|  |  | **Article 11**  2. In particular, the measures referred to in paragraph 1 shall include;    (b) facilitating the expansion of existing or the establishment of new capacities related to activities, the introduction of measures ensuring regulatory flexibility, aimed at supporting the production and placing on the market of crisis-relevant medical countermeasures;  [**Management of Medical Countermeasures**] |  |  |
|  |  | **Article 11**  2. In particular, the measures referred to in paragraph 1 shall include;    (c) implementing procurement initiatives, reserving stockpiles and production capacities to coordinate approaches, and providing critical supply, services and resources for the production of crisis-relevant medical countermeasures;  [**Management of Medical Countermeasures**] |  |  |
|  |  | **Article 11**  2. In particular, the measures referred to in paragraph 1 shall include;    (e) facilitating the licensing of intellectual property and know-how pertaining to the crisis-relevant medical countermeasures.  **[Management of Medical Countermeasures]** |  |  |
