# Supplementary material for: The architecture of the European Union’s pandemic preparedness and response policy framework
Source: Eur J Public Health. 2022 Nov 18;33(1):42–8. doi: 10.1093/eurpub/ckac154 (PMC9898003; doi:10.1093/eurpub/ckac154)
Supplement: ckac154_Supplementary_Data [file ckac154_supplementary_data.zip › ckac154_Supplementary_Data/ejph-2022-06-om-0333-File007.docx]

**Existing Competences (Pre adoption of Health Union proposals)**

| **Public Health Emergency Preparedness capability** | **Competence / Functionality** | **Institution** | **Source** |
| --- | --- | --- | --- |
| Detection & Assessment | The ECDC can be requested to carry out risk assessments to determine the potential severity of threats to the health of the public | European Centre for Disease Prevention and Control | Decision 1082/2013/EU |
| Detection & Assessment | Upon request, the ECDC can mobilize investigation and public health teams | European Centre for Disease Prevention and Control | Regulation 2004/851/EC |
| Detection & Assessment | The ECDC can also provide advice on the creation of case definitions but ultimately the Early Warning and Response Committee housed under the European Commission is responsible for adopting these definitions through implementing acts (Decision 1082/2013/EU) | European Centre for Disease Prevention and Control | Decision 1082/2013/EU |
| Detection & Assessment | The ECDC systematically collects, analyses and disseminates surveillance data on communicable diseases | European Centre for Disease Prevention and Control | (Scholz, 2020b) |
| Detection & Assessment | The ECDC encourages cooperation between laboratories across the EU to foster the development of capacity for diagnosis, detection and characterization of infectious agents | European Centre for Disease Prevention and Control | (Scholz, 2020b) |
| Policy Development, Adaptation & Implementation | The ECDC produces scientific opinions, guidelines, and recommendations | European Centre for Disease Prevention and Control | (Scholz, 2020b) |
| Healthcare services | The EU can deploy assistance and medical personnel through the Union Civil Protection Mechanism (UCPM), which forms the framework for cooperation allowing for Member States to pool together rescue teams and resources to be deployed for support in the field during disasters | European Commission | (European Commission, 2021z; Brooks et al., 2020; Scholz, 2020a) |
| Healthcare services | The EU European Medical Corps (EMC), can assist Member States by sending medical personnel when national health system capacity is overwhelmed (Haussig et al., 2017) | European Commission | (Haussig et al., 2017) |
| Coordination and Communication | Through high level structures such as the HSC and the IPCR, the EU Member States inform one another about epidemiological updates and response measures (Scholz, 2020a; De Miguel Beriain et al., 2015) | Health Security Committee / IPCR / European Commission | (Scholz, 2020a; De Miguel Beriain et al., 2015) |
| Emergency Risk Communication | A report of a Joint ECDC-ASEF Emergency Risk Communication workshop identified that the ECDC already completed several projects on Emergency Risk Communication including projects on community engagement in preparedness, the integration of emergency risk communication into national preparedness plans as well as strengthening country-targeted partnerships for emergency risk communication and preparedness under the new IHR framework | European Centre for Disease Prevention and Control | (ECDC, 2016) |
| Emergency Risk Communication | A report reviewing the ECDC actions during the COVID-19 pandemic highlighted the communication group within the ECDC, which is responsible for external communication with the press and media as well as for monitoring anxieties and rumors among Member States | European Centre for Disease Prevention and Control | (ECDC, 2021b). |

Supplementary Table 5: Existing EU Competences in Pandemic Preparedness and response (Pre adoption of European Health Union Legislative package)

| **Proposed Competences under European Health Union** | | | |
| --- | --- | --- | --- |
| **Public Health Emergency Preparedness capability** | **Competence / Functionality** | **Institution** | **Source** |
| Detection & Assessment | Both the EMA and HERA also have a role to play in epidemiological surveillance as the EMA would monitor the epidemiological situation to anticipate possible medicine shortages, and HERA would perform the same function but with an angle to identify avenues for which medical countermeasures need to be developed | EMA / HERA | (Article 2, Commission Decision establishing HERA, 2021; Article 4, Regulation EU 2022/123) |
| Detection & Assessment | The proposed regulation on Serious Cross Border Threats to Health (SCBTH) establishes an **advisory committee on public health emergencies** which would advise the Commission whether a threat constitutes a public health emergency at the Union level. | Advisory Committee on Public Health | Article 24, Proposal for a regulation on SCBTH) |
| Detection & Assessment | Effects of **recognition of a Public Health Emergency at Union level are extended to** activate mechanisms to monitor shortages of medical countermeasures as well as launch processes to develop, procure and deploy medical countermeasures. The recognition would also activate support from the ECDC to mobilise and deploy the EU health taskforce. | Commission / Advisory Committee on Public Health | (Article 25, proposal for a regulation on SCBTH) |
| Policy Development, Adaptation & Implementation | An EU-Wide pandemic preparedness and response plan is to be established | ECDC / Member States / European Commission | (Article 5, Proposal for a regulation on SCBTH) . |
| Policy Development, Adaptation & Implementation | European Commission may complement action of Member States through **adoption of recommendations on common temporary public** health measures for Member States | European Commission | (Article 22, Proposal for a regulation on SCBTH) |
| Policy Development, Adaptation & Implementation | HSC and ECDC can adopt n**on-binding opinions and guidance** on specific response measures for the European Union and its Member States | ECDC / Health Security Committee | (Article 3, Proposal ammending the the regulation EC No 851/2004 establishing the ECDC; Article 4 (2d) Proposal for a regulation on SCBTH) |
| Policy Development, Adaptation & Implementation | The ECDC can audit Member States on the implementation of their preparedness and response plans as well as their overall coherence with the EU preparedness plan | ECDC / Member States | (Article 8, Proposal for a regulation on SCBTH) |
| Policy Development, Adaptation & Implementation | the ECDC can propose **recommendations** and Member States would have to present an **action plan addressing the proposed recommendations and the corresponding corrective actions and milestones** | ECDC / Member States | (Article 8 (2), Proposal for a regulation on SCBTH) |
| Policy Development, Adaptation & Implementation | The new EMA emergency taskforce would help in the development of policies for treatment guidance and infection control by **providing scientific recommendations regarding the use of medicines to address public health emergencies** | EMA | (Article 15, Regulation 2022/123) |
| Policy Development, Adaptation & Implementation | The EMA is now tasked with **coordinating studies on the effectiveness and safety of medicinal products used to diagnose**, treat or prevent diseases related to a public health emergency (Article 20, Regulation 2022/123) | EMA | (Article 20, Regulation 2022/123) |
| Healthcare services | An “EU Health Taskforce” is established which would assist the local response to outbreaks of communicable diseases in Member States and third countries. | ECDC | Article 11a of the Regulation amending Regulation 2004/851 |
| Healthcare services | New provisions prohibit Member States from running parallel negotiation processes for medical countermeasures during the Joint Procurement Procedure | Member States / Commission | (Article 12 (2c), Proposal for a regulation on SCBTH) |
| Healthcare services | The Commission will ensure the coordination and information exchange during the Joint Procurement Procedure. | European Commission | (Article 12 (3), Proposal for a regulation on SCBTH) |
| Healthcare services | EMA’s Emergency Taskforce (ETF) will provide advice on clinical trial protocols and provide scientific support to facilitate and accelerate clinical trials for pandemic relevant medicinal products | EMA | (Article 15 & Article 16, Regulation 2022/123; Anderson et al., 2021; Mauer et al., 2022) |
| Healthcare services | The ETF will review scientific data on medical products which have the potential to be used to address a public health emergency | EMA | (Article 18, Regulation 2022/123; Mauer et al., 2022) |
| Healthcare services | The new Executive Steering Group on Shortages and Safety of Medicinal products (MSSG), and the Executive Steering Group on Shortages of Medical Devices (MDSSG) are responsible for establishing lists of critical medicines and medical devices whose stocks and availability are to be monitored following the recognition of a public health emergency | EMA | (Article 6, Regulation 2022/123). |
| Healthcare services | The steering groups can provide recommendations to the Commission or to Member States on action for the quality, safety, and efficacy of the medicinal products concerned as well as on existing shortages of critical medicine | EMA | (Article 5, Regulation 2022/123). |
| Healthcare services | In its preparedness mode, HERA is promoting research and development of medical countermeasures and related technologies,production of medical countermeasures and stockpiling capacity of medical countermeasures. | HERA | (Art 2, Commission Decision establishing HERA, 2021; Anderson et al., 2021; Article 2, Commission Decision establishing HERA, 2021; Mauer et al., 2022) |
| Healthcare services | In so-called “crisis mode”, HERA mandate would, among other things, include the monitoring, procurement, purchase and manufacturing of crisis relevant medical countermeasures (European Commission, 2021z) | HERA | (European Commission, 2021z) |
| Coordination and Communication | The Health Security Committee (HSC) is divided into two working formations, with one high-level working group to discuss topics of political importance as well as decisions on the adoption of opinions and guidance, and technical working groups to discuss specific topics of technical nature | Health Security Committee | (Article 4, Proposal for a regulation on SCBTH) |
| Coordination and Communication | The proposed SCBTH also formalizes cooperation between the ECDC and the HSC, with the former producing and disseminating regular reports on outbreaks of communicable diseases as well as providing outbreak forecasts. | Health Security Committee / ECDC | (Article 3(2), Proposal ammending the the regulation EC No 851/2004 establishing the ECDC; Article 5, Proposal ammending the the regulation EC No 851/2004 establishing the ECDC; Article 5b, Proposal ammending the regulation EC No 851/2004) |
| Emergency Risk Communications | This is further supported by Member State reporting requirements including the disclosure of risk communication capacities | Member States | (Article 7(b,ii) Proposal on a regulation for SCBTH) |
| Emergency Risk Communications | The advisory committee would also provide advice on response including advice on risk and crisis communication to be addressed to all Member States | Advisory Committee on Public Health | (Article 24 (1c,i), Proposal for a regulation on SCBTH) |
| Emergency Risk Communications | The ECDC is tasked with providing the HSC with evidence-based communication messages to the public | ECDC | (Article 3(2), Proposal ammending the the regulation EC No 851/2004 establishing the ECDC) |

Supplementary Table 6: foreseen EU Competences in Pandemic Preparedness and response ( Commission proposals on European Health Union)
