# Supplementary figures and images for: The architecture of the European Union’s pandemic preparedness and response policy framework
Source: Eur J Public Health. 2022 Nov 18;33(1):42–8. doi: 10.1093/eurpub/ckac154 (PMC9898003; doi:10.1093/eurpub/ckac154)

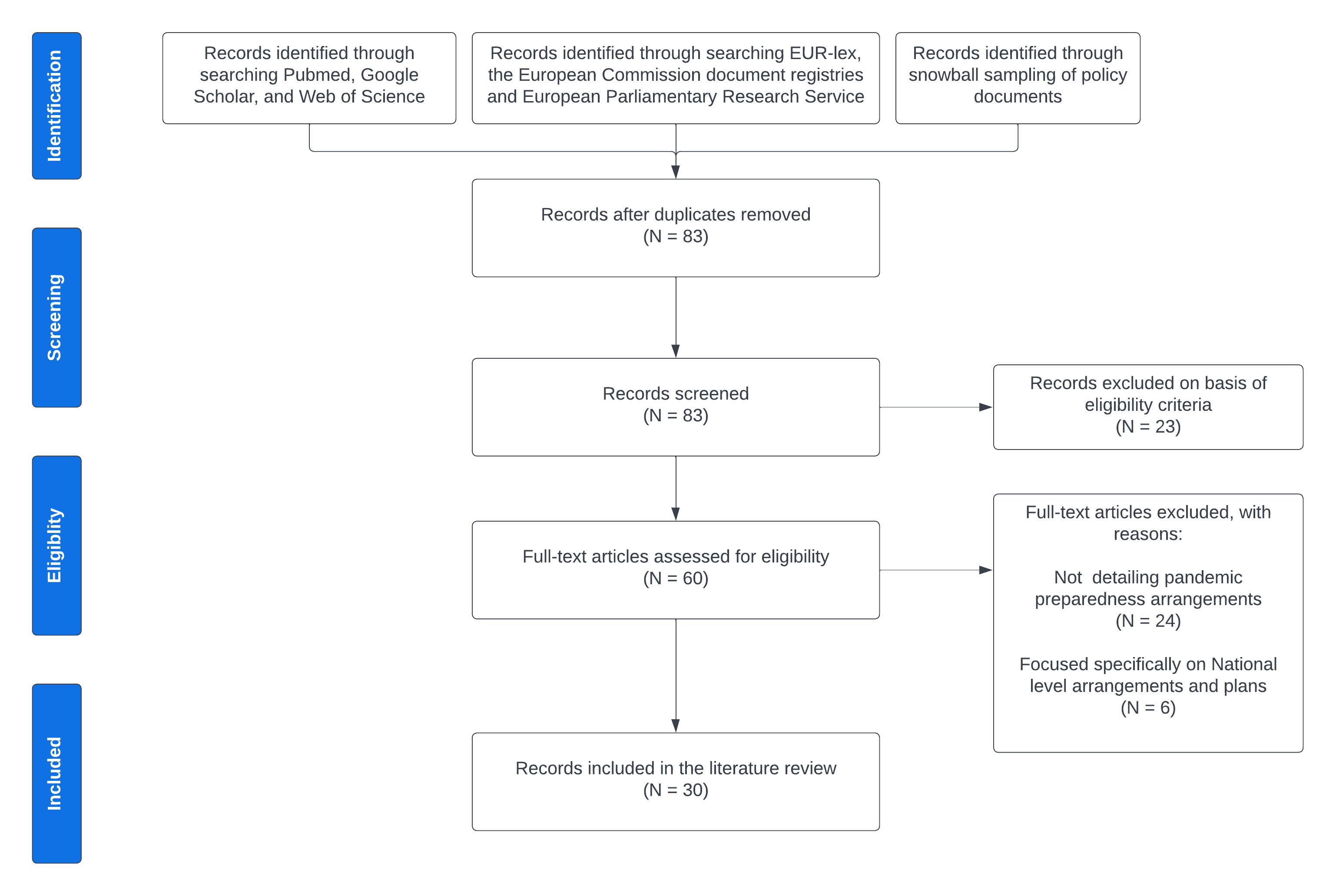

Supplement: ckac154_Supplementary_Data [file ckac154_supplementary_data.zip › ckac154_Supplementary_Data/ejph-2022-06-om-0333-File008.tiff]
